# Etiology-Related Differences in Left Ventricular Remodeling and Left Atrial Function in Patients with Heart Failure and Reduced Ejection Fraction Referred for ICD/CRT Implantation

**DOI:** 10.3390/jcm15186989

**Published:** 2026-09-09

**Authors:** Martyna Dąbrowska, Magdalena Potapowicz-Krysztofiak, Marek Kiliszek, Zbigniew Orski, Magdalena Smalc-Stasiak, Małgorzata Banak, Małgorzata Maciorowska, Paweł Krzesiński, Beata Uziębło-Życzkowska

**Affiliations:** Department of Cardiology and Internal Diseases, Military Institute of Medicine—National Research Institute, 04-141 Warsaw, Poland

**Keywords:** heart failure with reduced ejection fraction, non-ischemic cardiomyopathy, ischemic cardiomyopathy, left atrial strain, global wasted work, impedance cardiography

## Abstract

**Background:** Heart failure with reduced ejection fraction (HFrEF) may be caused by various factors. The etiology of HFrEF may influence prognosis and further management. **Objectives:** In this study we aimed to assess differences between ischemic and non-ischemic etiology in the context of clinical features, hemodynamic evaluation, and left ventricular (LV) and left atrial (LA) remodeling in a selected cohort of patients referred for device implantation. **Methods:** This prospective single-center study included 86 patients with HFrEF in sinus rhythm, without a history of atrial fibrillation, referred for ICD or CRT implantation. All patients underwent comprehensive echocardiography, electrocardiography, impedance cardiography and laboratory testing. This was a cross-sectional analysis without clinical follow-up. The pre-specified question was whether the two etiological groups differed in LV remodeling and LA function. All regression analyses were exploratory. Guideline-directed medical therapy was recorded in all patients. **Results:** Among 86 patients, 58 had ischemic and 28 had non-ischemic HFrEF. Patients with ischemic etiology were older, had worse renal function, and had higher N-terminal pro–B-type natriuretic peptide levels. They also exhibited lower left ventricular mass index (LVMI), left atrial reservoir strain (LASr), left atrial emptying fraction (LAEF), and higher left atrial stiffness index (LASI). Among myocardial work parameters, only global wasted work (GWW) differed significantly, with higher values in the non-ischemic group. Patients with ischemic HFrEF also had a higher Heather index (HI) in impedance cardiography. The two groups did not differ in guideline-directed medical therapy. In univariable logistic regression, older age, lower LVMI, reduced LASr and LAEF, higher LASI, higher HI, and lower eGFR were associated with ischemic HFrEF etiology. In the multivariable model, older age, lower LVMI, and reduced LASr remained independently associated with ischemic HFrEF (area under the ROC curve 0.82). **Conclusions:** Patients with ischemic and non-ischemic HFrEF referred for device implantation differ in LV remodeling and LA function. The between-group differences were small in absolute terms, with widely overlapping distributions, and the study was not designed to test whether echocardiography can replace established methods of establishing HFrEF etiology. These exploratory findings are hypothesis-generating and require confirmation in larger, independent cohorts.

## 1. Introduction

Heart failure (HF) is a chronic and heterogeneous syndrome, characterized by high morbidity, mortality and impaired quality of life. The prevalence of HF is estimated at 1–3% of the general population, reaching over 10% in patients aged over 80 years [1]. Left ventricular ejection fraction (LVEF) plays a pivotal role in HF diagnosis, prognosis and treatment indications. It is estimated that heart failure with reduced ejection fraction (HFrEF) accounts for approximately 50% of HF cases [2,3]. Despite significant advances in the treatment of HFrEF, which have led to improvements in symptoms, quality of life and clinical outcomes, the prognosis of HFrEF patients remains unfavorable, with a 5-year mortality rate of approximately 50% after diagnosis [4,5]. Nevertheless, LVEF should not be treated as the sole factor differentiating HF patients, as establishing HF etiology may also influence prognosis and further personalized management [4,6]. Coronary artery disease (CAD) remains the principal cause of HFrEF, followed by non-ischemic dilated cardiomyopathy, hypertension and other causes [7]. Different pathomechanisms underlie HF development depending on HF etiology (e.g., ischemia, inflammation, toxins, and genetics); however, the distinctions between ischemic HFrEF and non-ischemic HFrEF are still not fully understood. Cardiac magnetic resonance (CMR), by assessing the pattern of late gadolinium enhancement, is the imaging modality of choice for differentiating between ischemic and non-ischemic etiologies [8,9], although its main limitations are high cost and limited availability in standard clinical settings. Therefore, in our study we aimed to assess differences between ischemic and non-ischemic HFrEF in the context of clinical features, hemodynamic evaluation and detailed echocardiographic assessment of left ventricular (LV) and left atrial (LA) remodeling.

## 2. Materials and Methods

### 2.1. Data Collection

In this prospective single-center study, consecutive adult patients with symptomatic HFrEF (left ventricular ejection fraction (LVEF) ≤ 40%), with sinus rhythm and with no prior history of AF, were enrolled between July 2021 and December 2025 at the time of referral for ICD/CRT implantation, irrespective of HF etiology. The patients were qualified for device implantation in accordance with current guideline recommendations [10]. Exclusion criteria included a history of atrial arrhythmias, chronic obstructive pulmonary disease in stage E (former C or D) GOLD (Global Initiative for Chronic Obstructive Lung Disease) classification [11], significant mitral or aortic stenosis, and pregnancy. All participants underwent laboratory testing, electrocardiography (ECG) and echocardiography directly before device implantation. Estimated glomerular filtration rate (eGFR) was calculated using the Cockcroft–Gault equation (mL/min), which estimates creatinine clearance.

Chronic pharmacological treatment of heart failure at the time of enrollment was recorded for every patient from the hospital medication chart, including drug class and daily dose.

The etiology of HF was established before any of the analyses reported here by the treating cardiologist, on the basis of the clinical history, coronary angiography and echocardiography, supplemented by cardiac magnetic resonance in patients in whom the cause of LV dysfunction remained unclear. Cardiac magnetic resonance was not part of the study protocol. It was ordered by the treating physician when clinically indicated, and the pattern of late gadolinium enhancement, together with the accompanying tissue characterization, was used to support or to exclude a specific non-ischemic etiology. Endomyocardial biopsy was not performed in any patient. Ischemic HFrEF was defined as LV dysfunction attributable to coronary artery disease: a history of myocardial infarction, previous percutaneous coronary intervention or coronary artery bypass grafting, or angiographically significant stenosis of an epicardial coronary artery, to an extent judged sufficient to explain the degree of LV dysfunction. The presence of coronary artery disease alone was not considered sufficient for this diagnosis. Patients with limited coronary disease that did not explain the observed LV dysfunction—for example, single-vessel disease treated with elective angioplasty, without infarction and without a matching regional wall motion abnormality—were classified as non-ischemic. Non-ischemic HFrEF was defined as LV dilatation with systolic dysfunction not explained by abnormal loading conditions or coronary artery disease, that is, dilated cardiomyopathy. Every non-ischemic patient met this definition. Within this group the underlying trigger was recorded separately as post-inflammatory, alcohol-related (toxic), or not identified, in which case the cardiomyopathy was classified as idiopathic.

### 2.2. Study Objective

This was a cross-sectional, descriptive analysis of baseline data collected before device implantation. No clinical outcome was assessed, and no follow-up was performed. The study therefore has no primary or secondary endpoint in the sense used for trials or prognostic studies. The study addressed one pre-specified question: whether patients with ischemic and non-ischemic HFrEF referred for ICD or CRT implantation differ in LV remodeling and LA function. Echocardiographic assessment of the LV and the LA was the principal method used to answer it. All comparisons of impedance cardiography, myocardial work and laboratory variables—and all regression analyses—were exploratory; no correction for multiple testing was applied.

### 2.3. Ethical Statement

This study was conducted in accordance with the Declaration of Helsinki. All patients included in the study provided written informed consent. The study protocol was approved by the Ethics Committee of the Military Institute of Medicine—National Research Institute (Registration No. 23/2021, approval date 16 June 2021).

### 2.4. Echocardiographic Assessment

All echocardiographic examinations were performed using a commercially available ultrasound system (Vivid E95, GE Healthcare, Horten, Norway) in accordance with current ASE and EACVI recommendations [12,13]. Standard 2D, Doppler, and 2D-STE datasets were acquired.

Left ventricular (LV) volumes and LVEF were assessed using the biplane Simpson method from apical four- and two-chamber views. Diastolic function was evaluated based on transmitral flow parameters (E, A, E/A ratio) and tissue Doppler-derived mitral annular e’ velocities, with the E/e’ ratio used as an estimate of LV filling pressure [14].

Left ventricular mass was calculated from linear measurements according to the Devereux formula and indexed to body surface area to obtain the left ventricular mass index (LVMI).

Left atrial volume was measured from apical four- and two-chamber views and indexed to body surface area (LAVI). LA strain (LAS) was assessed using 2D speckle tracking echocardiography (2D STE) with QRS gating, providing reservoir (LASr), conduit (LAScd), and contraction (LASct) strain components, in line with EACVI/ASE consensus recommendations [15]. The LA stiffness index (LASI) was calculated as the ratio of early diastolic mitral inflow velocity to mitral annular velocity (E/e’) divided by LASr [16].

Left ventricular longitudinal strain was evaluated with 2D-STE in all apical chamber views, and global longitudinal strain (GLS) was calculated as the mean peak systolic strain across all 17 LV segments. Myocardial work parameters were estimated using non-invasive pressure–strain loop analysis, with brachial systolic pressure serving as a surrogate for peak LV pressure. Derived indices included global work index (GWI), global constructive work (GCW), global wasted work (GWW), and global work efficiency (GWE), following the methodology originally proposed by Russell et al. and later validated in several large-scale studies [17,18]. All post-processing was conducted offline using the EchoPAC software (version 204, GE Healthcare, Horten, Norway). Longitudinal and left atrial strain values were reported as absolute magnitudes.

### 2.5. Impedance Cardiography

Impedance cardiography was performed non-invasively before device implantation to assess the hemodynamic profile based on changes in thoracic electrical bioimpedance during the cardiac cycle, using a Niccomo™ device (Medis, Ilmenau, Germany). The following parameters were derived: stroke index (SI), stroke volume (SV), cardiac index (CI), systemic vascular resistance index (SVRI), velocity index (VI), thoracic fluid content (TFC), total arterial compliance index (TACI), and the Heather index (HI). The Heather index, an index of left ventricular contractility, was defined as the ratio of the maximum of the first derivative of the transthoracic impedance signal, (dZ/dt)max, to the time interval from the onset of the ECG Q wave to (dZ/dt)max [19,20].

Offline echocardiographic (including strain and myocardial work) and impedance cardiography analyses were performed by investigators blinded to heart failure etiology.

### 2.6. Statistical Analysis

Statistical analysis was performed using standard methods appropriate for the distribution and type of variables. Continuous variables are presented as medians with interquartile ranges, whereas categorical variables are presented as counts and percentages where applicable. Distribution normality was assessed before comparative analyses. All analyses were performed on complete cases; no imputation was used. The number of available measurements is given for every variable in Table 1 and Table 2. The main variables were complete or nearly complete (LVMI, LASr, LAEF and Heather index 86/86; LASI 85/86; eGFR 85/86; GWW 84/86), whereas hyperlipidemia status was available in 83/86, QRS duration in 78/86, NT-proBNP in 71/86, and the presence of left bundle branch block in 59/86 patients.

Because most analyzed variables did not meet the assumptions of normal distribution, comparisons between patients with ischemic and non-ischemic etiology were performed using the Mann–Whitney U test for continuous variables and the chi-square test or Fisher’s exact test, as appropriate, for categorical variables. A two-sided *p*-value below 0.05 was considered statistically significant.

To further assess the relationship between selected variables and ischemic etiology, logistic regression analysis was performed with ischemic etiology as the dependent variable. All odds ratios therefore describe the odds of ischemic rather than non-ischemic etiology. The mirror model with non-ischemic etiology as the dependent variable is provided in Supplementary Table S1. Because there are only two groups, it is the same model with inverted odds ratios. First, univariable logistic regression models were constructed for the variables that differed between groups. Odds ratios (ORs) with 95% confidence intervals (CIs) were calculated. Subsequently, variables showing significant associations in univariable analysis and considered clinically meaningful were entered into a multivariable logistic regression model to identify factors independently associated with ischemic etiology. To preserve an adequate number of events per variable, the multivariable model was restricted to age and the most robust echocardiographic predictors (LVMI and LASr). In a two-group logistic model, the limiting number is the size of the smaller group; with 28 non-ischemic patients and three covariates, this corresponds to 9.3 events per variable. For the same reason, the additional analyses described below were run as separate models rather than as one large model. Because the left atrial measures (LASr, LAEF, and LASI) are mathematically and physiologically interrelated, LASr was selected as the representative left atrial parameter to avoid collinearity. The results are reported as ORs with 95% CIs. Given the exploratory nature of the analyses and the number of comparisons performed, no adjustment for multiple testing was applied, and the findings should be regarded as hypothesis-generating. The discrimination of the multivariable model was assessed by the area under the ROC curve. Four additional sensitivity analyses were performed: additional adjustment for diabetes mellitus and hyperlipidemia; additional adjustment for guideline-directed medical therapy; additional adjustment for device type (CRT versus ICD); and restriction to the age range shared by both groups. In addition, GWW and each variable that was significant in univariable analysis but not included in the main model were tested in separate models together with age (Supplementary Table S2). The association between GWW and QRS duration was assessed with the Spearman rank correlation coefficient, and the independent association of etiology with GWW was tested in a linear regression model containing etiology, QRS duration and LVMI.

All statistical analyses were performed using Statistica 13.0 (TIBCO Software Inc., Palo Alto, CA, USA).

## 3. Results

### 3.1. Study Population

This study included 86 patients with HFrEF. Of these, 58 patients had ischemic etiology, and 28 had non-ischemic etiology. All 28 non-ischemic patients met the definition of dilated cardiomyopathy. The underlying trigger was post-inflammatory in 14 patients (50.0%) and alcohol-related (toxic) in three (10.7%); in 11 patients (39.3%), no trigger was identified, and their cardiomyopathy was classified as idiopathic. Thirty-three patients (38.4%) received an implantable cardioverter–defibrillator only, 50 (58.1%) a CRT-D and three (3.5%) a CRT-P; the proportion receiving a resynchronization device did not differ significantly between groups (55.2% vs. 75.0%, *p* = 0.099). Most patients were in NYHA class I or II, with no significant difference between groups (NYHA III or IV: 17 [29.3%] vs. 7 [25.0%], *p* = 0.790).

### 3.2. Baseline Clinical and Laboratory Differences

Patients with ischemic HFrEF were significantly older than those with non-ischemic HFrEF (68.0 vs. 59.5 years, *p* = 0.004; Table 1). They also had worse renal function, reflected by higher creatinine concentrations (1.20 vs. 1.10 mg/dL, *p* = 0.021) and lower eGFR values (63.3 vs. 81.2 mL/min, *p* = 0.002). N-terminal pro–B-type natriuretic peptide (NT-proBNP) concentrations were significantly higher in the ischemic group (2067.0 vs. 1047.5 pg/mL, *p* = 0.023). Hyperlipidemia was significantly more frequent in patients with ischemic HFrEF (48 [85.7%] vs. 17 [63.0%], *p* = 0.025), whereas diabetes mellitus was more frequent without reaching statistical significance (26 [44.8%] vs. 6 [21.4%], *p* = 0.056).

Guideline-directed medical therapy was recorded in all 86 patients and did not differ between groups. A beta-blocker was used in 56 (96.6%) versus 28 (100%) patients; an angiotensin-converting enzyme inhibitor, angiotensin receptor blocker or angiotensin receptor-neprilysin inhibitor in 56 (96.6%) versus 28 (100%); a mineralocorticoid receptor antagonist in 55 (94.8%) versus 27 (96.4%); and a sodium-glucose co-transporter 2 inhibitor in 48 (82.8%) versus 26 (92.9%). Complete four-drug therapy was given to 45 (77.6%) versus 25 (89.3%) patients (*p* = 0.246). The only differences concerned aspirin (54 [93.1%] vs. 15 [53.6%], *p* < 0.001) and statins (57 [98.3%] vs. 24 [85.7%], *p* = 0.037), which follow directly from the presence of coronary artery disease. Detailed treatment data are presented in Supplementary Table S5.

### 3.3. Etiology-Related Differences in Left Ventricular Remodeling and Left Atrial Function

Patients with ischemic etiology had significantly lower LVMI than those with non-ischemic etiology (137.6 vs. 158.7 g/m^2^, *p* = 0.014; Table 2). Global longitudinal strain did not differ significantly between groups (7.4 vs. 8.6%, *p* = 0.160). Left ventricular dimensions and volumes (LVDd, LVEDVi, and LVESVi), as well as LVEF, did not differ significantly between groups. The prevalence of moderate/significant mitral regurgitation did not differ significantly between groups (ischemic: 15 [25.9%] vs. non-ischemic: 8 [28.6%]; *p* = 0.800; Table 2).

Left atrial function differed significantly between etiological groups. Patients with ischemic HFrEF had lower LASr (16.0 vs. 17.0%, *p* = 0.021) and lower LAEF (40.0 vs. 45.0%, *p* = 0.021), indicating reduced reservoir and emptying function. These differences, although statistically significant, were modest in magnitude with overlapping distributions. In parallel, LASI was significantly higher in the ischemic group (0.80 vs. 0.62, *p* = 0.025), suggesting greater left atrial stiffness.

### 3.4. Additional Myocardial Work and Hemodynamic Findings

Among myocardial work parameters, GWW differed significantly between groups (312.0 vs. 381.0 mmHg%, *p* = 0.021), with higher indices in non-ischemic HFrEF, whereas no significant between-group differences were observed for GCW, GWI, or GWE. Among impedance cardiography-derived parameters, patients with ischemic HFrEF had a significantly higher HI than those with non-ischemic etiology (8.3 vs. 6.8, *p* = 0.011). Cardiac index showed only a borderline difference between groups (2.65 vs. 2.50 L/min/m^2^, *p* = 0.063). In univariable logistic regression, higher HI was significantly associated with ischemic etiology, whereas cardiac index did not emerge as a significant correlate.

Clinical, electrocardiographic and laboratory characteristics by HFrEF etiology are presented in Table 1. Echocardiographic characteristics and impedance-cardiography-derived parameters are presented in Table 2.

**Table 1:** Clinical, electrocardiographic, and laboratory characteristics according to HFrEF etiology.

Variable	Ischemic HFrEF		Non-Ischemic HFrEF		*p*
	***n***		***n***		
Clinical characteristics, median (IQR) or n (%)					
Age, years	58	68.0 (63.0–75.0)	28	59.5 (53.0–70.0)	0.004
BMI, kg/m^2^	58	26.1 (24.2–29.1)	28	26.5 (24.5–28.6)	0.724
Sex, male	58	49 (84.5)	28	23 (82.1)	0.760
Hypertension	58	43 (74.1)	28	15 (53.6)	0.080
Diabetes mellitus	58	26 (44.8)	28	6 (21.4)	0.056
Hyperlipidemia	56	48 (85.7)	27	17 (63.0)	0.025
Stroke	58	6 (10.3)	28	3 (10.7)	0.960
CKD	58	15 (25.9)	28	3 (10.7)	0.160
Alcohol	58	3 (5.2)	27	3 (11.1)	0.380
Smoking	57	28 (49.1)	27	14 (51.9)	0.810
COPD	58	9 (15.5)	28	2 (7.1)	0.490
Hypothyroidism	58	7 (12.1)	28	3 (10.7)	0.860
Electrocardiographic data, median, IQR					
HR, beats/min	53	68.0 (61.0–78.0)	26	70.5 (58.0–80.0)	0.897
P, ms	51	116.0 (104.0–130.0)	26	109.0 (100.0–128.0)	0.316
PQ, ms	52	177.0 (164.0–200.0)	26	170.5 (158.0–186.0)	0.067
QRS, ms	52	140.0 (110.0–160.0)	26	157.0 (116.0–170.0)	0.161
LBBB, n (%)	36	18 (50.0)	23	15 (65.2)	0.292
Laboratory findings, median, IQR					
Creatinine, mg/dL	57	1.20 (1.00–1.50)	28	1.10 (0.95–1.20)	0.021
eGFR, mL/min	57	63.3 (45.3–76.4)	28	81.2 (66.7–94.6)	0.002
NT-proBNP, pg/mL	47	2067.0 (940.0–3654.0)	24	1047.5 (532.9–2753.5)	0.023

Abbreviations: BMI, body mass index; CKD, chronic kidney disease; COPD, chronic obstructive pulmonary disease; eGFR, estimated glomerular filtration rate; HR, heart rate; IQR, interquartile range; NT-proBNP, N-terminal pro–B-type natriuretic peptide; LBBB, left bundle branch block; P, P wave duration; PQ, PQ interval; QRS, QRS duration.

**Table 2:** Echocardiographic characteristics and impedance-cardiography-derived parameters according to HFrEF etiology. Variables not directly related to the study question are presented in Supplementary Table S4.

Variable	Ischemic HFrEF		Non-Ischemic HFrEF		*p*
	***n***		***n***		
Echocardiographic data, median (IQR) or n (%)					
LVDd, mm	58	63.6 (59.8–68.1)	28	63.9 (61.4–71.5)	0.306
LVMI, g/m^2^	58	137.6 (120.9–167.6)	28	158.7 (132.7–188.9)	0.014
LASr, %	58	16.0 (9.0–20.0)	28	17.0 (14.5–21.0)	0.021
LASct, %	58	11.0 (5.0–15.0)	28	13.0 (9.0–16.0)	0.069
LAScd, %	58	4.0 (3.0–6.0)	28	5.0 (3.0–7.0)	0.396
LASI (E/e’/LASr)	57	0.80 (0.56–1.86)	28	0.62 (0.43–0.85)	0.025
LAEF, %	58	40.0 (27.0–48.0)	28	45.0 (40.0–50.5)	0.021
LVEDVi, mL/m^2^	54	89.6 (74.0–115.8)	28	95.9 (79.3–127.5)	0.177
LVESVi, mL/m^2^	54	61.6 (49.1–82.1)	28	68.3 (53.4–88.8)	0.190
SV (echocardiographic), mL	57	53.0 (47.0–63.0)	28	56.5 (43.5–65.0)	0.714
GCW, mmHg%	56	985.5 (824.5–1228.5)	28	1029.5 (899.5–1293.5)	0.294
GWW, mmHg%	56	312.0 (208.5–419.5)	28	381.0 (259.5–570.5)	0.021
GWI, mmHg%	56	680.5 (548.0–827.0)	28	676.0 (566.0–909.0)	0.447
GWE, %	56	76.0 (70.5–84.0)	28	76.0 (65.0–79.5)	0.243
LAVI, mL/m^2^	57	44.0 (36.8–58.6)	28	41.7 (37.5–50.2)	0.686
GLS LV, %	57	7.4 (6.0–9.1)	28	8.6 (6.5–10.0)	0.160
E/e’ avg	56	13.0 (9.9–20.0)	28	11.3 (9.2–14.0)	0.072
E/A	56	0.68 (0.55–1.30)	28	0.62 (0.51–0.81)	0.165
LVEF, %	57	32.0 (27.0–35.0)	28	29.0 (26.0–33.5)	0.290
MR ≥ moderate, n (%)	58	15 (25.9)	28	8 (28.6)	0.800
Impedance cardiography, median, IQR					
CI, L/min/m^2^	58	2.65 (2.40–3.00)	28	2.50 (2.15–2.75)	0.063
HI	58	8.3 (6.3–11.0)	28	6.8 (5.0–8.1)	0.011

Abbreviations: A, late diastolic transmitral inflow velocity; CI, cardiac index; E, early diastolic transmitral inflow velocity; E/A, ratio of early (E) to late (A) diastolic transmitral inflow velocities; E/e′ avg, ratio of early diastolic transmitral inflow velocity (E) to the average early diastolic mitral annular velocity (e′), measured by tissue Doppler imaging; GCW, global constructive work; GLS LV, left ventricular global longitudinal strain; GWE, global work efficiency; GWI, global work index; GWW, global wasted work; HI, Heather index; IQR, interquartile range; LAEF, left atrial emptying fraction; LAScd, left atrial strain during conduit phase; LASct, left atrial strain during contraction phase; LASI, left atrial stiffness index (E/e′/LASr); LASr, left atrial strain during reservoir phase; LAVI, left atrial volume index; LVDd, left ventricular end-diastolic dimension; LVEDVi, left ventricular end-diastolic volume index; LVEF, left ventricular ejection fraction; LVESVi, left ventricular end-systolic volume index; LVMI, left ventricular mass index; MR, mitral regurgitation; SV, stroke volume measured by echocardiography.

### 3.5. Uni- and Multivariable Logistic Regression Analysis

In univariable logistic regression, older age increased the likelihood of ischemic etiology (OR 1.070, 95% CI 1.022–1.121, *p* = 0.004). Among echocardiographic parameters, lower LVMI (OR 0.834, 95% CI 0.737–0.951 (per 10 g/m^2^), *p* = 0.007), reduced LASr (OR 0.897, 95% CI 0.823–0.978, *p* = 0.014), lower LAEF (OR 0.947, 95% CI 0.906–0.989, *p* = 0.015), and higher LASI (OR 2.622, 95% CI 1.123–6.122, *p* = 0.026) were significant correlates of ischemic disease (Table 3). In addition, a higher HI (OR 1.175, 95% CI 1.006–1.373, *p* = 0.042) and lower eGFR (OR 0.974, 95% CI 0.956–0.993, *p* = 0.007) were linked to the ischemic phenotype. GWW was not significantly associated with ischemic etiology in univariable analysis (OR 0.981, 95% CI 0.958–1.004 (per 10 mmHg%), *p* = 0.096). Although NT-proBNP differed significantly between groups, it was not associated with etiology in logistic regression (log-transformed NT-proBNP OR 1.450, 95% CI 0.966–2.177 per unit of natural logarithm, *p* = 0.073; n = 71; Table 3).

**Figure 1:**
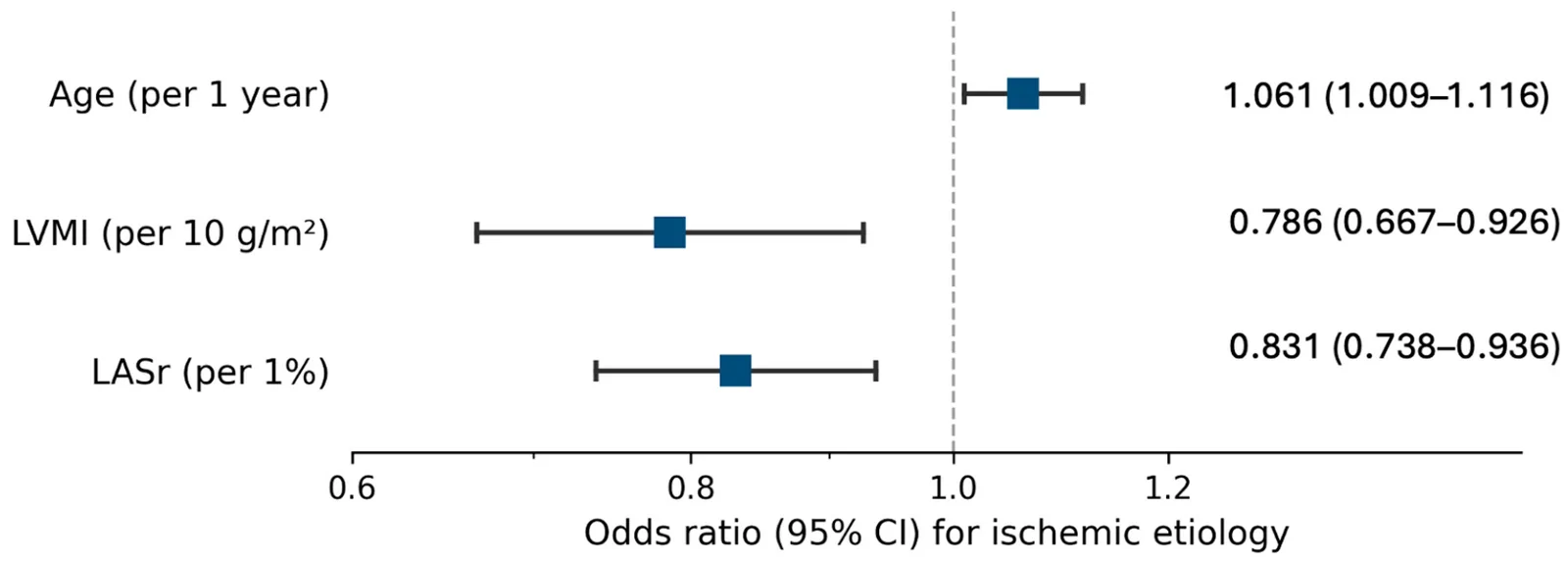
Forest plot of the multivariable logistic regression model (age, LVMI, and LASr). Points indicate odds ratios, and horizontal lines denote 95% confidence intervals. The dashed vertical line indicates an odds ratio of 1 (no association). LVMI was scaled per 10 g/m^2^.

In the multivariable model, older age (OR 1.061, 95% CI 1.009–1.116, *p* = 0.021), lower LVMI (OR 0.786, 95% CI 0.667–0.926 (per 10 g/m^2^), *p* = 0.004), and reduced LASr (OR 0.831, 95% CI 0.738–0.936, *p* = 0.002) remained independently associated with ischemic etiology (Figure 1). Neither GWW nor eGFR remained independent after adjustment for age. The model showed good discrimination (AUC 0.82; Table 3). In a sensitivity analysis additionally adjusted for diabetes mellitus and hyperlipidemia (n = 83), lower LVMI (OR 0.794, 95% CI 0.669–0.944 (per 10 g/m^2^), *p* = 0.009) and reduced LASr (OR 0.850, 95% CI 0.754–0.958, *p* = 0.008) remained independently associated with ischemic etiology, whereas neither comorbidity was independently associated with etiology (diabetes mellitus *p* = 0.300; hyperlipidemia *p* = 0.290). Because eGFR and HI were significant in the univariable analysis, and because GWW differed between groups even though it did not reach significance in the univariable analysis, each of these three variables was tested in a separate model together with age (Supplementary Table S2). None remained independently associated with etiology: GWW OR 0.973 (95% CI 0.947–1.000) per 10 mmHg%, *p* = 0.051; eGFR OR 0.985 (95% CI 0.963–1.007), *p* = 0.184; HI OR 1.109 (95% CI 0.948–1.298), *p* = 0.194. In contrast, the two remaining left atrial measures remained significant after adjustment for age: LAEF OR 0.943 (95% CI 0.898–0.989), *p* = 0.016, and LASI OR 2.723 (95% CI 1.114–6.655), *p* = 0.028. Apart from LV mass, left atrial function was therefore the only domain that distinguished the two etiologies independently of age.

The results were consistent across the remaining sensitivity analyses (Supplementary Table S3). After additional adjustment for guideline-directed medical therapy, lower LVMI (OR 0.785 per 10 g/m^2^, 95% CI 0.665–0.927, *p* = 0.004) and lower LASr (OR 0.836, 95% CI 0.742–0.941, *p* = 0.003) remained independently associated with ischemic etiology, whereas treatment itself was not (*p* = 0.244). After additional adjustment for device type, lower LVMI (OR 0.791, *p* = 0.006) and lower LASr (OR 0.824, *p* = 0.003) were likewise preserved. When the analysis was restricted to the age range shared by both groups (53–75 years, n = 58), lower LVMI (OR 0.813, *p* = 0.038) and lower LASr (OR 0.823, *p* = 0.005) remained significant, while age itself did not (*p* = 0.297), as expected when the age range is restricted by design.

**Figure 2:**
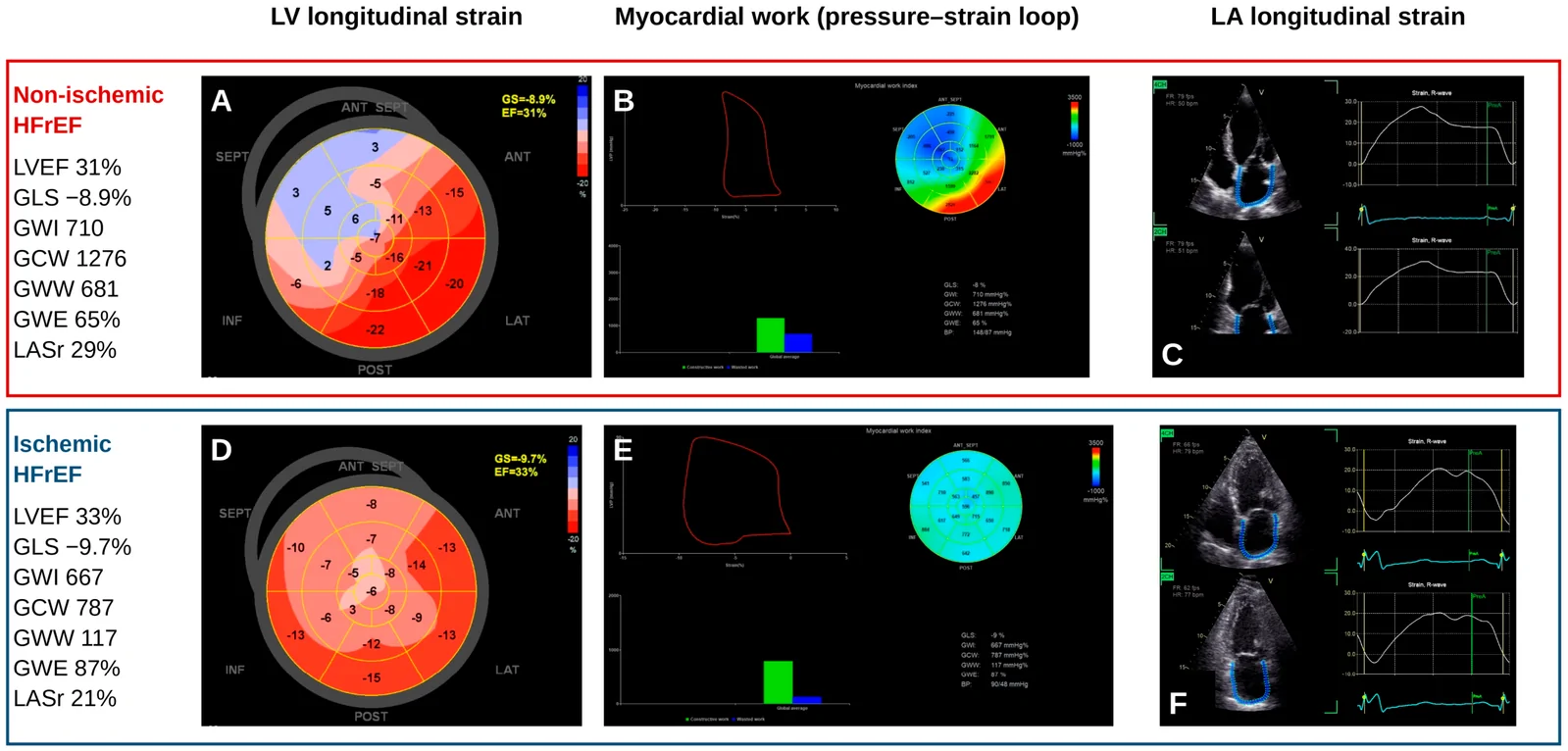
Representative examples of left ventricular longitudinal strain, myocardial work and left atrial strain in two patients with HFrEF and comparable left ventricular ejection fraction. GWI, GCW and GWW are expressed in mmHg%. Upper panels (**A**–**C**): a patient with non-ischemic HFrEF (dilated cardiomyopathy with left bundle branch block; LVEF 31%, GLS 8.9%, GWI 710 mmHg%, GWW 681 mmHg%). Lower panels (**D**–**F**): a patient with ischemic HFrEF (occlusion of the left anterior descending artery; LVEF 33%, GLS 9.7%, GWI 667 mmHg%, GWW 117 mmHg%). Panels (**A**,**D**) show a bull’s-eye display of global and regional left ventricular longitudinal strain; panels (**B**,**E**) show non-invasive pressure–strain loop analysis with a bull’s-eye display of the segmental myocardial work index and the corresponding global constructive and wasted work; panels (**C**,**F**) show left atrial longitudinal strain with its reservoir, conduit and contraction components. Strain values are shown as generated by the analysis software; that is, with their sign, in the text and tables, they are reported as absolute magnitudes. At a similar ejection fraction and global longitudinal strain, wasted work is several times higher in the patient with a left bundle branch block, in whom the septal segments stretch instead of shortening during systole, whereas in the ischemic patient, myocardial work is more uniformly and less severely reduced.

Examples of left ventricular longitudinal strain, myocardial work, and left atrial strain in two patients with HFrEF and comparable left ventricular ejection fractions are shown in Figure 2.

## 4. Discussion

The principal finding of this study is that ischemic and non-ischemic HFrEF represent distinct structural and functional phenotypes. In the multivariable model adjusted for age, LVMI and LASr remained independently associated with ischemic etiology, and the model showed good discrimination (AUC 0.82). In other words, ischemic HFrEF was characterized primarily by more impaired left atrial function, whereas non-ischemic HFrEF was characterized by greater LV mass-related remodeling. Several other between-group differences—worse renal function, higher NT-proBNP, higher Heather index and lower GWW—paralleled the older age and greater comorbidity burden of the ischemic group; none of them were independently associated with etiology, either because they were not significant in regression analysis (NT-proBNP, GWW) or because they did not persist after adjustment for age (eGFR, Heather index). This underscores that age is a major determinant of the ischemic phenotype in this population and that only LVMI and LA reservoir function distinguished the two etiologies independently of it.

Lower LVMI in ischemic etiology may be explained by myocardial scar and severe ischemic injury that promote ventricular dilatation, wall thinning, and impaired recovery of systolic function, ultimately leading to HF [21]. It should be noted, however, that in the present cohort, LV dimensions and volumes were comparable between groups, so the lower LVMI in ischemic patients reflects a lower calculated LV mass at a similar cavity size rather than more pronounced dilatation. In contrast, non-ischemic HFrEF is characterized by myocyte apoptosis and excessive collagen deposition, leading to diffuse interstitial fibrosis [22]. Moreover, although no patient in the present cohort had hypertrophic cardiomyopathy, left ventricular hypertrophy may, paradoxically, lead to systolic dysfunction and development of HFrEF. This phenomenon is well documented in end-stage hypertrophic cardiomyopathy, a condition characterized by advanced heart failure with reduced LVEF [23,24]. These patients may represent a different pattern of LV remodeling, driven by microvascular dysfunction and gradual LV wall fibrosis [25]. These findings are in line with other studies using CMR indices. Wang et al., in their retrospective observational study, compared ischemic cardiomyopathy with non-ischemic cardiomyopathy and coexisting incidental myocardial infarction using CMR [26]. Patients with non-ischemic cardiomyopathy and incidental myocardial infarction showed more pronounced LV dilatation despite having similar LVEF to those with ischemic cardiomyopathy.

Left atrial remodeling occurs frequently in HFrEF and is characterized by structural and mechanical chamber modifications. Left atrial strain globally reflects atrial function, remodeling, and distensibility and is progressively reduced in the setting of LV dysfunction. In the present study, patients with ischemic HFrEF had worse left atrial function, with lower LASr and LAEF and higher LASI. Similarly, Sharifov et al. reported that patients with CAD with significant occlusions had decreased LAEF compared with those without CAD and that LASr decreased in CAD with significant occlusions compared with CAD with moderate occlusions [27]. Notably, that study was conducted on a small sample of patients without HF, yet it indicated that impairment of left atrial function is driven by CAD and its hemodynamic consequences, even in the absence of increased left ventricular filling pressures. Apart from reflecting left ventricular systolic and diastolic dysfunction, left atrial strain parameters may identify individuals at increased risk of adverse cardiovascular outcomes. The majority of existing studies present them in the context of outcome stratification, whereas hemodynamic evaluation is scarcely mentioned [28,29,30]. Together with the multivariable results, this positions impaired LA reservoir function as one of the two features that distinguish ischemic HFrEF independently of age.

Taken together, these findings suggest that non-ischemic HFrEF was characterized by greater LV-mass-related remodeling, whereas ischemic HFrEF was more strongly associated with functional impairment of the left atrium. The absolute differences were nevertheless small, and the distributions overlapped widely (LASr 16.0% vs. 17.0%; LAEF 40% vs. 45%), so these parameters cannot be used to assign etiology in an individual patient. What the findings indicate is that the structural consequences of the underlying disease mechanism are not limited to the left ventricle and may involve different patterns of atrioventricular interaction.

Patients with ischemic HFrEF were also older and had worse renal function, but this difference should be interpreted in the context of age. According to ESC registry data, patients with ischemic HF are older (median 67 vs. 58 years) and have higher LVEF (28 vs. 25%) than those with non-ischemic etiology [31]. Consistently, in a large comparative study of chronic HF patients, Smilde et al. found that patients with non-ischemic dilated cardiomyopathy were younger and had better renal function than those with ischemic etiology [32]. Mechanisms underlying renal dysfunction in HF include hemodynamic changes (low cardiac output, increased venous congestion), inflammatory and neurohormonal activation, and drug-induced impairment [33], which are common to both etiologies. In our cohort, although eGFR was lower in the ischemic group in univariable analysis, it did not remain independently associated with etiology after adjustment for age. This suggests that the poorer renal function of ischemic patients largely reflects their older age and greater comorbidity burden rather than an etiology-specific effect, although ischemia-related contributors, such as microvascular damage and multi-vessel atherosclerosis with significant stenoses of the renal arteries, may have additional effects.

A similar pattern was seen for myocardial work. Among myocardial work indices, patients with ischemic HFrEF had lower GWW than those with non-ischemic HFrEF, whereas GCW, GWI and GWE did not differ. GWW reflects work that does not contribute to cardiac output and increases with adverse LV geometry and congestion [34]. Physiologically, wasted work is generated by segments that shorten out of phase with the rest of the ventricle, through early systolic stretch and post-systolic shortening, and the amount of myocardium available to waste work is larger when LV mass is larger. A lower GWW accompanying a lower LVMI is therefore expected rather than paradoxical. Consistently, Sahiti et al. showed that higher LV mass and volume are associated with greater wasted work [35]. The second determinant of wasted work is mechanical dyssynchrony. In our cohort, GWW showed a weak, non-significant association with QRS duration (Spearman rho 0.215, *p* = 0.062), and the non-ischemic group had a numerically wider QRS (157 vs. 140 ms, *p* = 0.161) and a more frequent left bundle branch block (65.2% vs. 50.0%, *p* = 0.292), suggesting a greater degree of electrical dyssynchrony. In a linear model containing etiology, QRS duration and LVMI, none of the three variables were associated with GWW (etiology *p* = 0.251, QRS duration *p* = 0.480, LVMI *p* = 1.000), and GWW was not independently associated with etiology after adjustment for age (*p* = 0.051). Accordingly, the lower GWW in ischemic HFrEF is unlikely to reflect superior contractile performance; in our cohort, the between-group difference was not explained by LV mass or QRS duration and did not persist after adjustment for age. It is therefore best regarded as a marker of LV geometry and dyssynchrony rather than an independent etiological discriminator.

The results from impedance cardiography indicated that the ischemic phenotype of HFrEF was associated with a higher HI, which reflects the mechanical efficiency of the heart as a pump [20]. However, this difference was not accompanied by a significant difference in LVEF or other impedance indices of the heart as a pump (CI, SI, and VI). Therefore, this result should not be interpreted as indicating superior contractility in ischemic HFrEF.

Overall, our findings indicate that HFrEF is an etiologically heterogeneous condition representing the final common pathway of myocardial damage. The etiology-dependent pattern of remodeling was not limited to morphological changes of the LV wall but also involved functional and hemodynamic consequences. Among these, only lower LV mass and impaired LA reservoir function distinguished ischemic from non-ischemic HFrEF independently of age. These observations were made in a selected population of device candidates, and the echocardiographic parameters were not compared with a uniform imaging reference standard since cardiac magnetic resonance was obtained only when it was clinically indicated; they should therefore be regarded as hypothesis-generating.

## 5. Limitations

This study has several limitations. The sample size was modest, especially in the non-ischemic subgroup, which may have limited statistical power and the stability of regression estimates. With 28 patients in the smaller group and three covariates, the multivariable model contained 9.3 events per variable, which is close to the conventional minimum, so the confidence intervals should be interpreted with caution. Although patients were enrolled prospectively, the analysis was cross-sectional and based on baseline assessment without follow-up, so causal inference cannot be made. In addition, patients with atrial fibrillation were excluded from the analysis and the results may not be extended to this population. The non-ischemic group was uniform in phenotype, since all 28 patients met the definition of dilated cardiomyopathy, but heterogeneous with respect to the underlying trigger, which was post-inflammatory in 14 patients, alcohol-related in 3 and not identified in 11. Whether these subgroups differ from one another in LV and LA remodeling could not be tested, because each is too small to support a multivariable model. Because HF etiology was established a priori from clinical, angiographic and, where indicated, cardiac magnetic resonance data, the regression models should be regarded as descriptive and hypothesis-generating rather than diagnostic. The two groups also differed in age and comorbidity burden, and although age was included in every model and a sensitivity analysis restricted to the shared age range gave consistent results, residual confounding cannot be excluded. In addition, the cohort comprised candidates for ICD or CRT implantation, i.e. a selected advanced heart failure population frequently with prolonged QRS, which may limit generalizability to unselected patients with HFrEF. 

The strengths of our study include the comparison of ischemic and non-ischemic HF etiologies within a previously unrepresented context of left ventricular and left atrial remodeling. To our knowledge, this is among the first studies to jointly characterize left atrial strain and myocardial work across HFrEF etiologies. The study employed novel imaging techniques, speckle tracking echocardiography and myocardial work. In the available literature, most studies comparing heart failure etiologies are retrospective analyses, and echocardiographic data are limited.

## 6. Conclusions

In this selected cohort of patients with HFrEF in sinus rhythm referred for ICD or CRT implantation, ischemic and non-ischemic etiologies were associated with different patterns of LV remodeling and LA function. Ischemic etiology was associated with lower LVMI, lower LASr, lower LAEF and higher LASI, indicating less pronounced LV mass-related remodeling but greater impairment of LA function. In multivariable logistic regression, older age, lower LVMI and lower LASr remained independently associated with ischemic etiology. The absolute differences were small, with widely overlapping distributions, and these exploratory findings do not support the use of echocardiographic phenotyping to determine etiology in an individual patient. They require confirmation in larger, independent cohorts with imaging-based verification of etiology.

## Figures and Tables

**Table 3:** Uni- and multivariable logistic regression analysis for ischemic etiology in HFrEF.

Variables	Univariable Analysis			Multivariable Analysis		
	*p*-Value	Odds Ratio (OR)	OR 95% CI	*p*-Value	Odds Ratio (OR)	OR 95% CI
Age, years	0.004	1.070	1.022–1.121	0.021	1.061	1.009–1.116
LVMI (per 10 units), g/m^2^	0.007	0.834	0.737–0.951	0.004	0.786	0.667–0.926
LASr, %	0.014	0.897	0.823–0.978	0.002	0.831	0.738–0.936
LASI	0.026	2.622	1.123–6.122			
LAEF, %	0.015	0.947	0.906–0.989			
GWW (per 10 units), mmHg%	0.096	0.981	0.958–1.004			
HI	0.042	1.175	1.006–1.373			
eGFR, mL/min	0.007	0.974	0.956–0.993			
NT-proBNP (log-transformed, per unit ln), n = 71	0.073	1.450	0.966–2.177			

Multivariable model: age + LVMI + LASr. In a two-group logistic model, the limiting number is the size of the smaller group: with 28 non-ischemic patients and 3 covariates, this corresponds to 9.3 events per variable. AUC = 0.82. Odds ratios for LVMI are expressed per 10 g/m^2^. Abbreviations: CI, confidence interval; eGFR, estimated glomerular filtration rate; GWW, global wasted work; HI, Heather index; LAEF, left atrial emptying fraction; LASI, left atrial stiffness index; LASr, left atrial strain during reservoir phase; LVMI, left ventricular mass index; NT-proBNP, N-terminal pro–B-type natriuretic peptide; OR, odds ratio.

## Data Availability

The data presented in this study are available upon request from the corresponding author due to privacy restrictions.

## Supplementary Materials

The following supporting information can be downloaded at https://www.mdpi.com/article/10.3390/jcm15186989/s1: Table S1: Multivariable logistic regression model with non-ischemic etiology as the dependent variable (mirror model); Table S2: Age-adjusted models for variables significant in univariable analysis but not included in the main multivariable model; Table S3: Sensitivity analyses of the main multivariable model; Table S4: Echocardiographic and impedance-cardiography variables not directly related to the study question; Table S5: Pharmacological treatment according to HFrEF etiology.
