# Supplementary material for: Etiology-Related Differences in Left Ventricular Remodeling and Left Atrial Function in Patients with Heart Failure and Reduced Ejection Fraction Referred for ICD/CRT Implantation
Source: J Clin Med. 2026 Sep 9;15(18):6989. doi: 10.3390/jcm15186989 (PMC13607521; doi:10.3390/jcm15186989)
Supplement: Supplementary file 1 [file jcm-15-06989-s001.zip › jcm-4520441-supplementary.pdf]

Supplementary Material

Supplementary Table S1. Multivariable logistic regression model with non-ischemic etiology as the dependent variable (mirror model)

Variable	Odds ratio	95% CI	p-value
Age, years	0.942	0.896–0.991	0.021
LVMI (per 10 g/m²)	1.272	1.080–1.499	0.004
LASr, %	1.203	1.068–1.354	0.002

n = 86. Because there are only two etiological groups, this model is identical to the main model reported in Table 3, with odds ratios inverted (OR here = 1/OR in Table 3). Abbreviations as in Table 3.

Supplementary Table S2. Age-adjusted models for variables significant in univariable analysis but not included in the main multivariable model

Variable	n	Odds ratio	95% CI	p-value
GWW (per 10 mmHg%)	84	0.973	0.947–1.000	0.051
eGFR, mL/min	85	0.985	0.963–1.007	0.184
Heather index	86	1.109	0.948–1.298	0.194
LAEF, %	86	0.943	0.898–0.989	0.016
LASI	85	2.723	1.114–6.655	0.028

Each variable was tested in a separate logistic regression model together with age, with ischemic etiology as the dependent variable. Only the left atrial measures (LAEF and LASI) remained significantly associated with etiology after adjustment for age.

Supplementary Table S3. Sensitivity analyses of the main multivariable model

Analysis	n	Age OR (p)	LVMI OR per 10 g/m² (p)	LASr OR (p)	AUC
Main model (Table 3)	86	1.061 (0.021)	0.786 (0.004)	0.831 (0.002)	0.823
+ diabetes mellitus and hyperlipidemia	83	1.062 (0.045)	0.794 (0.009)	0.850 (0.008)	0.843
+ guideline-directed medical therapy*	86	1.065 (0.017)	0.785 (0.004)	0.836 (0.003)	0.824
+ device type (CRT vs ICD)	86	1.082 (0.007)	0.791 (0.006)	0.824 (0.003)	0.833
Restricted to shared age range (53–75 years)	58	1.062 (0.297)	0.813 (0.038)	0.823 (0.005)	0.807

**Quadruple therapy (beta-blocker + ACEI/ARB/ARNI + MRA + SGLT2 inhibitor); treatment itself was not independently associated with etiology (p=0.244). All 28 non-ischemic patients met the definition of dilated cardiomyopathy, so no restriction to that diagnosis was possible; the main model is already the dilated cardiomyopathy analysis. Lower LVMI and lower LASr remained independently associated with ischemic etiology in every analysis. In the age-restricted analysis, age was no longer significant, as expected when the age range is narrowed by design. Abbreviations as in Tables 1–3.*

Supplementary Table S4. Echocardiographic and impedance-cardiography variables not directly related to the study question.

Variable	n (ischemic)	Ischemic HFrEF	n (non-ischemic)	Non-ischemic HFrEF	p

RVDd, mm	58	32.0 (30.0–34.0)	28	31.0 (28.5–34.5)	0.396
LVSd, mm	58	56.3 (49.9–61.9)	28	54.0 (51.0–63.0)	0.880
PSD, ms	56	102.5 (71.9–147.4)	28	103.4 (79.8–153.2)	0.388
LAa, cm²	57	24.6 (21.2–31.0)	28	23.9 (21.2–27.1)	0.359
RAa, cm²	57	15.5 (13.3–19.4)	28	16.2 (13.9–17.8)	0.632
TAPSE, mm	57	19.0 (15.0–21.0)	28	19.8 (16.5–21.6)	0.471
TRV, m/s	54	2.29 (2.03–2.90)	25	2.38 (2.10–2.52)	0.611
E, cm/s	56	51.5 (40.5–77.0)	28	43.5 (38.5–58.5)	0.146
A, cm/s	56	69.5 (52.5–79.0)	28	71.0 (57.5–87.0)	0.392
SI, ml/m²	55	37.6 (32.9–43.0)	28	35.6 (30.4–40.4)	0.095
SV (ICG), ml	55	71.0 (62.0–82.0)	28	66.0 (56.5–85.0)	0.217
VI	58	44.0 (37.0–56.0)	28	44.0 (36.5–56.0)	0.677
SVRI, dyn·s·cm^{-5}·m²	58	2311.0 (2004.0–2751.0)	28	2635.5 (2086.5–3011.0)	0.106
TACI	57	0.86 (0.69–1.10)	28	0.95 (0.65–1.00)	0.967
TFC	58	30.1 (27.9–35.5)	28	33.1 (28.4–36.9)	0.230
S’RV, cm/s	56	10.0 (8.0–12.0)	28	10.0 (8.0–11.5)	0.839
e’avg cm/s	56	4.0 (3.5–5.0)	28	4.5 (4.0–5.0)	0.146

Median (interquartile range). Ischemic n=58, non-ischemic n=28; the number of available measurements is given for each variable. None of these variables differed significantly between groups. A, late diastolic transmitral inflow velocity; E, early diastolic transmitral inflow velocity; e’avg, average early diastolic mitral annular velocity; LAa, left atrial area; LVSd, left ventricular end-systolic dimension; PSD, peak strain dispersion; RAa, right atrial area; RVDd, right ventricular end-diastolic diameter; SI, stroke index; S’RV, peak systolic velocity of the tricuspid annulus; SV, stroke volume; SVRI, systemic vascular resistance index; TACI, total arterial compliance index; TAPSE, tricuspid annular plane systolic excursion; TFC, thoracic fluid content; TRV, tricuspid regurgitation velocity; VI, velocity index.

Supplementary Table S5. Pharmacological treatment according to HFrEF etiology.

**Variable**	**n (ischemic)**	**Ischemic HFrEF**	**n (non-ischemic)**	**Non-ischemic HFrEF**	**p**
Beta-blocker	58	56 (96.6)	28	28 (100.0)	1.000
ACEI, ARB or ARNI	58	56 (96.6)	28	28 (100.0)	1.000
ACE inhibitor	58	17 (29.3)	28	7 (25.0)	0.800
ARNI	58	38 (65.5)	28	21 (75.0)	0.461
ARB	58	1 (1.7)	28	0 (0.0)	1.000
MRA	58	55 (94.8)	28	27 (96.4)	1.000
SGLT2 inhibitor	58	48 (82.8)	28	26 (92.9)	0.322
Quadruple therapy*	58	45 (77.6)	28	25 (89.3)	0.246
Loop diuretic	58	46 (79.3)	28	20 (71.4)	0.420
Statin	58	57 (98.3)	28	24 (85.7)	0.037
Aspirin	58	54 (93.1)	28	15 (53.6)	<0.001
Amiodarone	58	5 (8.6)	28	4 (14.3)	0.465
Digoxin	58	0 (0.0)	28	1 (3.6)	0.326

*n (%). *Quadruple therapy: beta-blocker + ACEI/ARB/ARNI + MRA + SGLT2 inhibitor. Ischemic n=58, non-ischemic n=28. Abbreviations: ACEI, angiotensin-converting enzyme inhibitor; ARB, angiotensin receptor blocker; ARNI, angiotensin receptor-neprilysin inhibitor; MRA, mineralocorticoid receptor antagonist; SGLT2, sodium-glucose co-transporter 2.*
